# Isolation of Cellulose Nanofibers from Kombucha Beverage By-Product by Chemo-Mechanical Routes

**DOI:** 10.3390/polym17172307

**Published:** 2025-08-26

**Authors:** Cătălina-Diana Uşurelu, Gabriela-Mădălina Oprică, Denis Mihaela Panaitescu, Adriana Nicoleta Frone, Celina Maria Damian, Cristian Andi Nicolae, Ştefan-Ovidiu Dima, Florin Oancea, Mircea Teodorescu

**Affiliations:** 1 National Institute for Research & Development in Chemistry and Petrochemistry—ICECHIM, 202 Splaiul Independentei, 060021 Bucharest, Romania; catalina.usurelu@icechim.ro (C.-D.U.); madalina.oprica@icechim.ro (G.-M.O.); cristian.nicolae@icechim.ro (C.A.N.); ovidiu.dima@icechim.ro (Ş.-O.D.); florino@ping.ro (F.O.); 2 Faculty of Chemical Engineering and Biotechnology, National University of Science and Technology POLITEHNICA, 1-7 Gh. Polizu Street, 011061 Bucharest, Romania; celina.damian@upb.ro (C.M.D.); mircea.teodorescu@upb.ro (M.T.)

**Keywords:** kombucha, cellulose nanofibers, fibrillation, purification

## Abstract

In a world where the negative consequences of natural resources’ overexploitation for the environment are increasingly evident, repurposing waste to obtain high-value goods becomes essential. This study proposes the isolation of cellulose nanofibers from the bacterial cellulose (BC) membrane that results as a by-product during the fermentation of Kombucha tea by chemical treatment with sodium hydroxide (NaOH), sodium hypochlorite (NaClO), hydrogen peroxide (H_2_O_2_), sulfuric acid (H_2_SO_4_) or citric acid, followed by mechanical fibrillation via high-speed homogenization and microfluidization. Treatments with NaOH, NaClO, and H_2_O_2_ were effective in the purification of Kombucha-derived BC, while H_2_SO_4_ and citric acid exhibited a rather weak cleaning action, as revealed by Fourier transform infrared spectroscopy and X-ray photoelectron spectroscopy. Besides their cleaning effect, the applied chemical pretreatments had an important effect on the degree of fibrillation attained, as indicated by the scanning electron microscopy images. This study proposes simple and effective routes to obtain bacterial cellulose nanofibers from an inexpensive and abundant source, commonly regarded as a waste material, which can be further applied in medical and packaging applications as reinforcing agents, adsorbent materials, or scaffolds.

## 1. Introduction

Reigning as nature’s most abundant organic polymer, cellulose is a material of great value in both industrial and research environments due to its inexpensiveness, excellent mechanical strength, biocompatibility, biodegradability, and the plenitude of hydroxyl groups from its structure which offer endless possibilities for physical and chemical modification. Particularly appealing are the nano forms of cellulose, collectively referred to as “nanocellulose” (NC), which can be isolated from cellulose by chemical, mechanical, enzymatic, or combined treatments [1], and are advantageous due to their light weight, higher specific surface area and aspect ratio, and greater Young’s modulus as compared to traditional cellulose [2]. Owing to their great availability, wood and cotton continue to be the primary sources for cellulose extraction [3]. However, using wood to produce cellulose heightens the risk of deforestation, which is associated with the loss of animal habitats, increased floods, climate change, and global warming [4]. Similarly, cotton cultivation requires land and large amounts of water that could be allocated to more important food crop cultivation [5]. As a result, in recent years, researchers have focused on the exploitation of agro-industrial lignocellulosic waste as an alternative source for the isolation of cellulose. While this can alleviate the problem of waste build-up in the environment [6], the agro-industrial waste typically exhibits lower cellulose content as compared to wood and cotton [7]. Additionally, the extraction of high-purity cellulose from vegetal sources, whether wood, cotton, or lignocellulosic waste, is challenging and laborious, involving a series of chemical treatments with reagents harmful to the environment and human health, and relatively harsh conditions to remove the hemicellulose, pectin, and lignin that closely accompany cellulose in plants [8].

An attractive alternative to plant-derived NC is bacterial cellulose (BC), a type of NC produced extracellularly by some species of bacteria, particularly from the *Komagataeibacter* genus, through the fermentation of various carbon sources [1]. Bearing the same molecular formula as its plant analogue, BC brings to the table a superior purity, higher degrees of polymerization, higher crystallinity, greater tensile strength [9] even in a wet state [10], higher water uptake and holding capacity, and a unique arrangement into a web-like three-dimensional network of ultrafine fibers [9]. Apart from its intended production, BC can result as a by-product during the obtaining of Kombucha beverages through the fermentation of tea broth using a symbiotic culture of bacteria and yeast (SCOBY) [11]. Kombucha is a fermented tea beverage whose popularity and consumption has increased in recent years due to its multiple beneficial properties, including its anti-inflammatory, antibacterial, antioxidant, anticancer, antihypertensive, and antidiabetic effects, as well as probiotic activity [12]. Traditionally, Kombucha tea is prepared via the aerobic fermentation of black tea and white sugar, at temperatures between 18 and 30 °C, for 7–15 days [13]. During the fermentation process, a multitude of compounds including acetic acid, gluconic acid, glucuronic acid, polyphenols, flavonoids, tannins, vitamins, minerals, D-saccharic acid-1,4-lactone, lipids, peptides, proteins, and enzymes [14] are generated, which provide the Kombucha beverage with various beneficial effects on human health.

The BC membrane produced by bacteria at the air–liquid interface during Kombucha tea fermentation is believed to play an important role in protecting bacteria from various stress factors (UV irradiation, elevated ethanol or acetic acid levels, hydrostatic pressure, etc.), retaining moisture to prevent bacteria dehydration, and ensuring the oxygen exposure required for fermentation [15]. Kombucha-derived BC (KBC) has a brown color, mainly due to melanoidin-type impurities, which are nitrogen-containing compounds formed by the Maillard reactions taking place between reducing sugars and amino acids or proteins during fermentation [11]. Other studies attribute the brown color of KBC to tannins in the tea broth [16]. Additionally, the KBC film contains entrapped yeast and bacteria cells, as well as other metabolites from the culture media, including amino acids, peptides, proteins, and oligosaccharides, which calls for effective methods of purification before use [11].

Despite being a rich source of cellulose, the KBC membrane is often regarded as a waste product of the kombucha beverage industry and discarded in the environment at the end of fermentation. However, in recent years, efforts have been made to capitalize on the cellulose from the Kombucha residue by processing it into high-value products such as supports for catalysts [17], materials for oral applications [18], scaffolds, wound dressings, adsorbents, leather substitutes [19], etc. Amarasekara et al. [20] investigated several protocols for the purification of KBC which consisted of two successive treatments with sodium hydroxide (NaOH) followed by bleaching with either hydrogen peroxide (H_2_O_2_) or sodium hypochlorite (NaClO) at room temperature. Alternatively, the same procedures were applied with the sole difference that the two NaOH treatments were conducted at 90 °C. From the proposed protocols, the method involving two treatments with NaOH at 90 °C followed by bleaching with NaClO was the most effective, leading to the KBC with the highest whiteness reading, and, therefore, the highest purity. This protocol was used and proven efficient in other works [21,22].

Bovi et al. [23] proposed three different procedures for the purification of KBC which involved thorough washing with distilled water, treatment with a 0.5 M NaOH solution at 70 °C for 4 h, or treatment with a 0.9 M potassium hydroxide (KOH) aqueous solution at room temperature for 14 h, each after extensive washing of the KBC with tap water. Of the three, the last method was the most convenient, leading to the apparently complete removal of the brown color of the KBC. Other works utilize simple treatment with a 1 M NaOH solution at 80 °C for 1 h followed by washing with water for purifying KBC [24,25]. In fact, most studies in the literature use treatment with aqueous NaOH solutions of different concentrations, at different temperatures and times, succeeded by washing with water, as a sole and effective method for purifying the KBC membrane [26]. When used as a purifying agent, NaOH acts by hydrolyzing the cellular debris, proteins, peptides, and other impurities in KBC with the formation of water-soluble or smaller-molecular-weight compounds that can be subsequently removed by washing or diffusion into water [27]. The removal of impurities from KBC is crucial for avoiding any immune response when KBC is used in biomedical applications [27].

The purified Kombucha membranes, consisting of a dense network of intertwined fibers, can be used as such or after surface modification in certain medical or industrial applications, but fibrillation of the membranes into individual fine fibers is necessary [17,21,28,29] when BC is used as reinforcing agent in composites—since it allows for better contact with the polymer matrix [30]—as an adsorbent [31], or in applications where transparency is required such as electronics, packaging, optics [21,32], etc. Dima et al. [11] concluded that 25 cycles of microfluidization at around 1300 bars of an aqueous suspension of KBC that had previously been purified with a 4 M NaOH solution and ground with a blender and then with a colloidal mill, resulted in the near-complete fibrillation of KBC into nanofibers, as demonstrated by the scanning electron microscopy (SEM) images. Pillai et al. [33] performed the nano-fibrillation of KBC via a chemical route that involved the partial acid hydrolysis of the KBC pellicle with 30% (*v*/*v*) sulfuric acid for 4 h at 70 °C, when they obtained a NC gel that could be used as ink for the 3D printing of scaffolds. Popa-Tudor et al. [22] showed that 20 microfluidization cycles of an aqueous suspension of KBC—previously purified by double treatment with NaOH for 1 h at 90 °C and then with NaClO at room temperature and ground with a blender—are sufficient to obtain an elevated degree of fibrillation, as evidenced by the transmission electron microscopy (TEM) images. KBC membranes previously purified by alkaline treatment were also nano-fibrillated by high-speed homogenization, and were used together with carboxymethyl cellulose in the production of bio-based active packaging materials [21].

The lack of fast and cheap purification procedures for this Kombucha beverage by-product and the lack of utilization chains cause these cellulose-rich KBC membranes to be discarded. Therefore, in this work, we studied the effect of several simple chemical pretreatments on the purification and activation of mechanical fibrillation of KBC membranes with the aim of promoting the use of nano-fibrillated KBC in the bionanocomposites sector. For this purpose, the KBC membranes, obtained as a by-product of Kombucha tea fermentation, were chemically treated with different agents, including NaOH, NaClO, H_2_O_2_, H_2_SO_4_, and citric acid, and the impact of the chemical pretreatments on both the purification and mechanical fibrillation of KBC was investigated in this work for the first time. Fourier transform infrared (FTIR) spectroscopy, X-ray photoelectron spectroscopy (XPS), X-ray diffraction (XRD), thermogravimetric analysis (TGA), polarized optical microscopy (POM), atomic force microscopy (AFM), and SEM were used to investigate the effects of the chemical pretreatments and mechanical fibrillation, via combined high-speed homogenization and microfluidization, on KBC’s properties. This work demonstrates that simple pretreatments using a single chemical agent can be effective for purifying KBC and enhancing its fibrillation.

## 2. Materials and Methods

### 2.1. Materials

In this study, the raw material consisted of a Kombucha membrane obtained using a SCOBY isolated from a Romanian culture and sweetened tea infusion [11]. Prior to use, the KBC membrane was thoroughly washed with distilled water. Citric acid (99.5%, Sigma-Aldrich, Steinheim, Germany), NaOH (pellets, 98%, Carl Roth, Karlsruhe, Germany), NaClO (15 *w*/*v*% in water, Scharlau, Barcelona, Spain), H_2_O_2_ (30 wt%, Chimreactiv SRL, Bucharest, Romania), and H_2_SO_4_ (95–97 wt%, Honeywell Fluka, Steinheim, Germany) were used as received.

### 2.2. Isolation of Cellulose Nanofibers from the Kombucha Membrane

**Figure 1:**
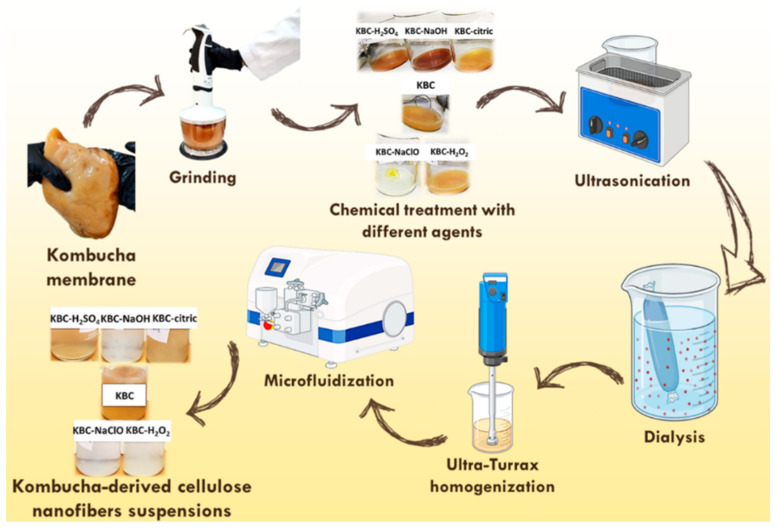
The sequential steps involved in the isolation of cellulose nanofibers from KBC (Created in BioRender. Usurelu, C.-D. (2025) https://BioRender.com/438zpab, accessed on 28 July 2025).

To isolate cellulose nanofibers from the Kombucha membrane, aqueous solutions of 5 wt% NaOH, 60 wt% H_2_SO_4_, and 60 wt% citric acid were prepared, while the NaClO and H_2_O_2_ aqueous solutions were used as received. Except for the 60 wt% citric acid solution, which was subjected to magnetic stirring at 80 °C and 500 rpm for 10 min, to ensure the complete dissolution of the citric acid in water, all other solutions were prepared at room temperature (RT). Next, the Kombucha membrane was subjected to grinding in a chopper for about 90 s. Subsequently, a certain volume from the 5 wt% NaOH, 15 *w*/*v*% NaClO, 30 wt% H_2_O_2_, 60 wt% H_2_SO_4_, or 60 wt% citric acid solution was added to 15 g of the ground Kombucha membrane suspension containing ~1 wt% solid, so that the weight ratio between the purifying solution and the solid in the Kombucha suspension was 100:1 in each case. The mixtures were left to stand at RT for 3 days and then subjected to ultrasonication at 75 °C for 3 h in an Elmasonic S40H ultrasonication bath (Elma, Singen, Germany) to potentiate the removal of impurities from the KBC. After sonication, the solid fraction contained by each mixture was isolated by centrifugation at 9000 rpm for 10 min, washed 3 times with 100 mL of distilled water in a centrifuge (9000 rpm/10 min), and finally recovered by centrifugation. The obtained solid was redispersed in 100 mL distilled water and subjected to dialysis using Spectra/Por^®^ membranes (Repligen, Waltham, MA, USA) with a molecular weight cut-off of 3500 Da, for 7 days, with daily change of the dialysis water until the pH remained constant. Next, the resulting aqueous suspensions of the treated Kombucha membranes were subjected to high-speed homogenization in a digital Ultra-Turrax T18 (IKA, Staufen, Germany) for 4 min at 13,000 rpm, and then to microfluidization at 1500 bar, for 10 cycles, in an LM20 microfluidizer (Microfluidics Inc., Westwood, MA, USA). The sequential steps involved in the isolation of cellulose nanofibers from KBC are presented in Figure 1.

Depending on the agent used for the chemical treatment, the resulting samples were labeled “KBC-NaOH”, “KBC-NaClO”, “KBC-H_2_O_2_”, “KBC-H_2_SO_4_”, and “KBC-citric”. For the sake of comparison, a control sample which was subjected to all the steps, except for the treatment with a chemical agent, was prepared and labeled “KBC”. Further, KBC-NaOH, KBC-NaClO, KBC-H_2_O_2_, KBC-H_2_SO_4_, and KBC-citric films were obtained by the deposition of small volumes from the aqueous suspensions of each sample on polystyrene dishes, followed by drying at 40 °C for 48 h and then again, at the same temperature, under vacuum, for 24 h.

### 2.3. Characterization

The chemical structure of the KBC films was investigated by FTIR spectroscopy, in the attenuated total reflectance mode, on a TENSOR 37 spectrometer (Bruker, Billerica, MA, USA). The FTIR spectra were acquired in the 4000–450 cm^−1^ range, at a resolution of 4 cm^−1^, each spectrum resulting from the average of 16 scans.

The surface chemical composition of the KBC films was investigated by XPS using a K-Alpha spectrometer (Thermo Scientific, Waltham, MA, USA) fitted with a monochromatic Al Kα source (1486.6 eV) operating at a vacuum base pressure of 2 × 10^−9^ mbar. The charging effects were neutralized using a flood gun and the binding energy was calibrated by assigning the C1s peak at 285 eV as the internal standard. The XPS survey spectra were recorded at a passing energy of 200 eV, while the high-resolution spectra were collected at a passing energy of 20 eV. The reported relative atomic concentrations from the XPS survey spectra represent the average of three measurements for each sample. The deconvolution of the high-resolution spectra into individual components was performed via Voigt curve fitting using Fityk software (version 1.3.1) [34].

The crystal structure of the KBC films was evaluated using a SmartLab diffractometer (Rigaku, Tokyo, Japan) with monochromatic CuKα radiation at a tube voltage of 45 kV and a tube current of 200 mA, over the 2θ range from 10 to 30°. Fityk software [34] was used for the analysis of the XRD diffractograms and deconvolution of the peaks, while the degree of crystallinity was calculated by dividing the area of crystalline peaks by that of crystalline and amorphous peaks. The interplanar distance and crystallite size were calculated using Bragg’s law and the Scherrer equation [28].

The thermal behavior of the KBC films was investigated by TGA. The analysis was conducted on a TA Q5000 thermobalance (TA Instruments Inc., New Castle, DE, USA), from 50 °C to 750 °C, at a constant heating rate of 10 °C/min, under an inert atmosphere of nitrogen, at a gas flow rate of 40 mL/min.

The efficiency of the Ultra-Turrax high-speed homogenization in the fibrillation of the KBC samples was assessed by POM utilizing an Olympus BX53F optical microscope provided with a DP23/DP28 Digital Camera (Olympus, Tokyo, Japan) at 10× magnification. The optical microscopy images were recorded using Olympus cellSens Entry software ver. 3.2 (Olympus, Tokyo, Japan) on samples prepared by depositing a volume of each KBC sample suspension on a microscope glass slide.

The degree of fibrillation after chemical treatment with different agents and mechanical treatments via high-speed homogenization and microfluidization was evaluated by SEM on sponges obtained by freeze-drying an aqueous suspension from each KBC sample, followed by coating with a 5 nm layer of gold using a Q150R Plus Sputter Coater (Quorum Technologies, Laughton, UK). The images were collected on a tabletop scanning electron microscope Hitachi TM4000 Plus II (Hitachi, Tokyo, Japan) at an accelerating voltage of 15 kV using the backscattered-electron detector.

More morphological details from the surface of the KBC films were obtained by atomic force microscopy (AFM) using a MultiMode 8 microscope from Bruker (Billerica, MA, USA) in Peak Force (PF) Quantitative Nanomechanical Mapping (QNM) mode at a scanning rate of 1.0 Hz. Silicon tips with a resonance frequency of 75 kHz and a nominal radius of 12 nm were used for this purpose.

## 3. Results

### 3.1. Fourier Transform Infrared Spectroscopy (FTIR)

**Figure 2:**
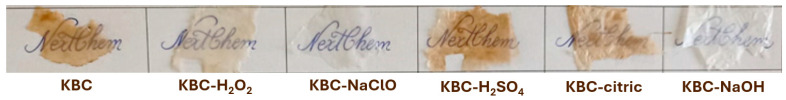
The aspect of the obtained KBC, KBC-H_2_O_2_, KBC-NaClO, KBC-H_2_SO_4_, KBC-citric, and KBC-NaOH films.

**Figure 3:**
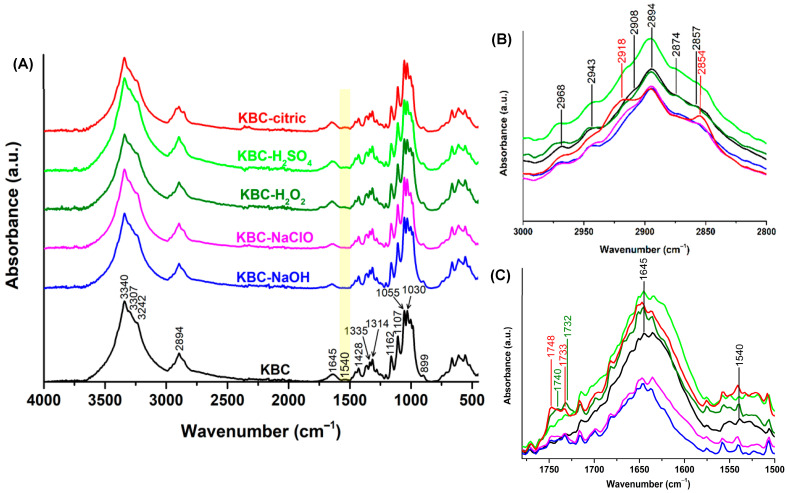
FTIR spectra of control KBC and KBC-NaOH, KBC-NaClO, KBC-H_2_O_2_, KBC-H_2_SO_4_, and KBC-citric samples in the 4000–450 cm^−1^ range (**A**) with insets in the 3000–2800 cm^−1^ domain (**B**) and 1780–1500 cm^−1^ domain (**C**); Insets share the same legend as Figure 3A.

Figure 2 reveals the aspect of the control KBC, KBC-H_2_O_2_, KBC-NaClO, KBC-H_2_SO_4_, KBC-citric, and KBC-NaOH films, while Figure 3 shows their corresponding FTIR spectra.

All FTIR spectra were normalized against the absorption at 1055 cm^−1^, which corresponds to the C-O-C stretching vibrations of the cellulose backbone [35]. The control KBC presents the bands characteristic of cellulose, while the contaminants that darken the film led to only minor changes in the FTIR spectra, as discussed below. The broad band from 3650 to 3000 cm^−1^ corresponds to the stretching vibrations of the inter- and intramolecularly hydrogen bonded OH groups in cellulose [36,37], with the peak at 3340 cm^−1^ being ascribed to the O3H group intramolecularly hydrogen bonded to the ring O5 atom, the one at 3307 cm^−1^ to the O6H group intermolecularly hydrogen bonded to the O3′ atom, and the one at 3242 cm^−1^ to the O6H group intramolecularly hydrogen bonded to the O2 atom [36,37] (Figure 3A). Actually, the peak at ~3240 cm^−1^ is characteristic of the cellulose Iα-allomorph [37], the dominant crystalline phase in BC.

The shoulders at 2968 and 2943 cm^−1^ may be assigned to the asymmetric C-H stretching vibrations of methylene (CH_2_) groups [38], the small shoulder at 2908 cm^−1^ and the intense peak at 2894 cm^−1^ to the asymmetric C-H stretching vibrations [39], and the two shoulders at 2874 and 2857 cm^−1^ to the symmetric C–H stretching vibrations [37] in KBC (Figure 3B). The band from 1780 to 1580 cm^−1^ and centered at 1645 cm^−1^, detailed in Figure 3C, is mainly assigned to the bending vibrations of the H-O-H bonds in absorbed water [40,41], but may include contributions from the amide I band (C=O stretching vibrations) [42] from proteins, peptides, or bacteria and yeast residues retained in KBC [43], as well as carbonyl (C=O), C=C, and imine (C=N) stretching vibrations or N-H bending vibrations from contaminants originating from the fermentation medium, such as flavonoids, tannins [44] (which are subclasses of polyphenolic compounds), and melanoidins [45]. Also, a minor but broad band situated between 1560 and 1510 cm^−1^ can be observed in the case of untreated KBC, and may be attributed either to the amide II vibrations (C-N stretching coupled with N-H bending), from proteins, peptides or bacterial residues [46] entrapped in KBC, or to potential melanoidin impurities [45].

Other important peaks are the ones at 1428 cm^−1^, corresponding to the CH_2_ bending vibrations and associated with the cellulose I crystalline phase in KBC [47]; 1335 cm^−1^, which can be assigned both to the C-H deformations and O-H in-plane bending vibrations; and 1314 cm^−1^, which can be ascribed to the CH_2_ out-of-plane wagging vibrations [48]. The peaks at 1162, 1107, 1055, and 1030 cm^−1^, respectively, are assigned to the C-O-C asymmetric stretching vibrations of the β-glycosidic linkages, C-C stretching/C-OH bending vibrations, skeletal C-O-C stretching vibrations of the pyranose ring [48], and C-O stretching [49]. The peak at around 899 cm^−1^ can be ascribed to the C-O-C stretching vibrations of the β-(1→4)-glycosidic bonds in cellulose and is characteristic of the cellulose I phase in KBC [48].

As shown in Figure 3C, treatments with NaOH and NaClO, respectively, resulted in a reduction and narrowing of the band centered at 1645 cm^−1^, and a major reduction of the broad band between 1560 and 1510 cm^−1^, along with a slight shift (of 1–2 cm^−1^) of the peaks at 1540 and 1537 cm^−1^, which are associated with the amide II band, to higher wavelengths, all events suggesting the removal of contaminants including flavonoids, tannins, melanoidins, peptides, proteins, and bacterial residues upon KBC’s treatment with NaOH or NaClO. This is consistent with the white color observed for KBC-NaOH and KBC-NaClO by visual inspection (Figure 2), which hints at the effective removal of the brown-colored contaminants from KBC and the obtaining of purer cellulose. In a previous study, Kashcheyeva et al. reported the disappearance of the peak at 1549 cm^−1^, attributed to the presence of proteins and cell residues, from the FTIR spectrum of a bacterial cellulose after purification with a 2% NaOH solution, a dilute HCl solution, and washing with distilled water until neutral pH was reached [50]. Furthermore, a reduced intensity of the 1620–1640 cm^−1^ band in the FTIR spectra of BC samples purified with non-conventional, biodegradable surfactants, compared to the untreated BC, was ascribed to protein contaminant removal [51].

From the faint brownish color of the KBC-H_2_O_2_ sample (Figure 2), treatment with H_2_O_2_ appears to also have led to a certain degree of cleaning of KBC, but not as much as in the case of NaOH and NaClO. Consequently, the absorption band at 1560–1510 cm^−1^ and centered at 1540 cm^−1^ is still visible in the FTIR spectrum of KBC-H_2_O_2_ (Figure 3C). Moreover, the appearance of two new peaks at 1740 and 1732 cm^−1^ (Figure 3C), which can be assigned to the stretching vibrations of the C=O bonds in aldehyde and/or carboxyl groups [52], suggests the oxidation of some of the C-OH groups in KBC following H_2_O_2_ treatment. The partial oxidation of the OH groups of cellulose with the formation of aldehyde and carboxyl groups due to the action of H_2_O_2_ assisted by a metal catalyst has been reported before [52]. Thus, it is possible that in the case of KBC-H_2_O_2_, the band centered at 1540 cm^−1^ also contains contributions from the asymmetric stretching vibrations of the newly formed carboxylate (COO^−^) groups [53] generated by the oxidation of KBC with H_2_O_2_. Interestingly, small shoulders can also be observed in the KBC-NaClO spectrum at these wavenumbers, indicating an oxidizing effect in the case of NaClO as well.

Citric acid does not appear to lead to an advanced cleaning of KBC, as evidenced by the band from 1560 to 1510 cm^−1^, which is still visible in the KBC-citric spectrum, as well as the darker color of the KBC-citric acid sample (Figure 2), which, however, differs from that of the untreated KBC. Treatment with citric acid seems to have brought about some changes in the chemical structure of KBC. Thus, as shown in Figure 3B, two new peaks appeared at 2918 cm^−1^ and 2854 cm^−1^ in the FTIR spectra of citric acid-treated KBC, which may be assigned to the asymmetric and symmetric C–H stretching vibrations of the CH_2_ groups in the citrate moieties that became covalently bound to the KBC fibers [54] during the treatment. Additionally, two new peaks could be observed at 1748 and 1733 cm^−1^, respectively, which can be ascribed to the C=O stretching vibrations of ester groups [54] or unreacted carboxyl groups [55] from the citrate moieties. These peaks indicate that the COOH groups of the citric acid may have participated in esterification reactions with the OH groups from KBC, with the formation of citrate groups bearing no, one, or two free carboxyl groups on its surface.

Similarly, treatment with H_2_SO_4_ does not appear to effectively clean KBC, the band at 1540 cm^−1^ remaining visible in this case. This is also confirmed by the deep-brown color of the KBC-H_2_SO_4_ sample, which is similar to that of untreated KBC (Figure 2). However, in the case of KBC-H_2_SO_4_, the high concentration of H_2_SO_4_ (60 wt%) and long treatment time (~72 h) may have led to KBC’s degradation, and hence the brown color of the final product. As is well known, H_2_SO_4_ causes the hydrolytic cleavage of the β-(1→4)-glycosidic bonds, primarily from the amorphous regions in cellulose, with the formation of degradation products of smaller molecular weight, which may exhibit a brown color and become immobilized onto KBC or become trapped within the water layer adsorbed at the cellulose surface [56]. Thus, it is possible that H_2_SO_4_ has acted more towards the acid hydrolysis of the C-O-C glycosidic bonds in cellulose, which are more susceptible to acid hydrolysis than the melanoidins, proteins, yeast and bacterial residues, and other impurities in KBC [57]. As a result, the FTIR spectrum of KBC-H_2_SO_4_ does not differ significantly from that of control KBC, except for the new shoulders appearing between 1765 and 1720 cm^−1^ (Figure 3C). These may be attributed to the C=O stretching vibrations from the aldehyde or carboxyl groups of potential humins, which are polymeric by-products that have been reported to form during the acidic hydrolysis of polysaccharides with H_2_SO_4_ [58].

### 3.2. X-Ray Photoelectron Spectroscopy (XPS)

**Figure 4:**
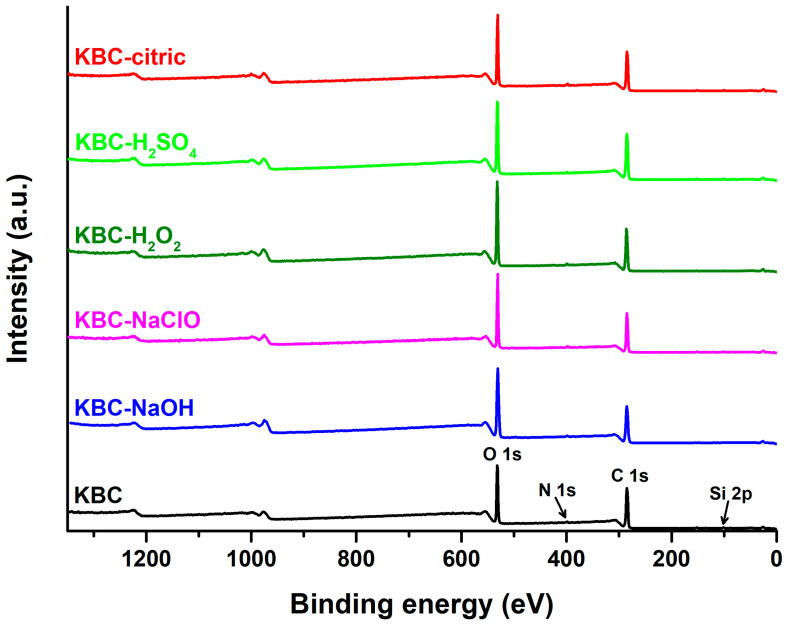
XPS survey spectra of the control KBC, KBC-NaOH, KBC-NaClO, KBC-H_2_O_2_, KBC-H_2_SO_4_, and KBC-citric samples.

**Figure 5:**
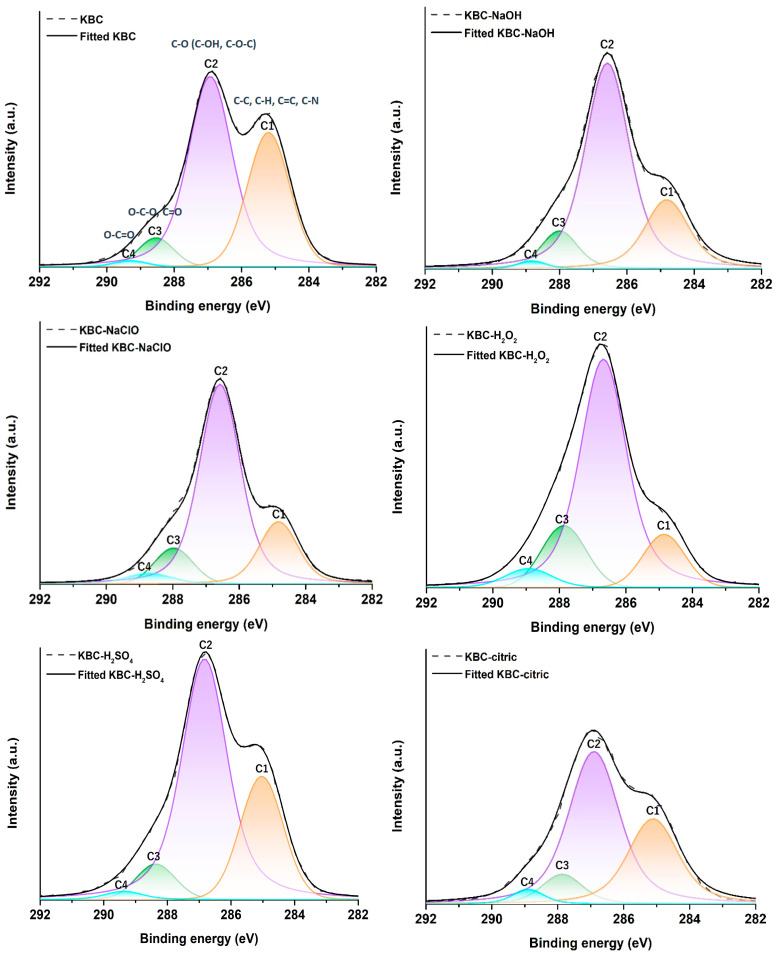
High-resolution XPS spectra of the C1s region with deconvolutions for KBC, KBC-NaOH, KBC-NaClO, KBC-H_2_O_2_, KBC-H_2_SO_4_, and KBC-citric.

XPS is a tool that allows semi-quantitative analysis of the elemental composition at the surface [59] of the resulting KBC films. The XPS survey and high-resolution spectra of the KBC films are displayed in Figure 4 and Figure 5, respectively, while the relative atomic percentages of elements and differently bound carbon atoms at the surface of the KBC samples are presented in Table 1 and Table 2, respectively. As shown in Figure 4, the survey spectra of control KBC showed characteristic peaks at 532 eV and 285 eV, attributed to the oxygen (O 1s) and carbon (C 1s) atoms, as well as at 399 eV and 101 eV, assigned to nitrogen (N 1s) and silicon (Si 2p) [60] atoms from impurities in KBC. While the peak from 399 eV can be attributed to the C-N, C=N, NH_2_, or -NH- groups [61] from peptides, enzymes, proteins, melanoidins, or yeast and bacteria residues in KBC, the peak at 101 eV may be assigned to the presence of adventitious Si originating from the glassware used during the chemical treatments or storage of the samples. However, both the N and Si contents are below 2%, indicating a relatively low level of such impurities in the samples [46].

The expected O/C atomic ratio in pure BC is 0.83 [60]. However, as shown in Table 1, a lower O/C value of 0.48 was recorded for control KBC, due to impurities attached to its surface [28,60], which are known to insert excess carbon into the material. For KBC-NaOH and KBC-NaClO, a significant increase in the O/C atomic ratio could be observed, from 0.48 to 0.65 and 0.6, respectively, confirming the efficiency of NaOH and NaClO treatments in the removal of impurities from KBC, in agreement with the FTIR results. H_2_O_2_ also had a pronounced purifying effect, leading to an increase in the O/C ratio to 0.58. This increase can also be attributed to the oxidizing action of H_2_O_2_, which can result in the formation of carboxyl groups in KBC, and, therefore, to a rise in the atomic percentage of O from the sample [59]. The lowest O/C increase was recorded for KBC-H_2_SO_4_, which suggests the poor ability of H_2_SO_4_ to remove impurities from KBC, as also indicated by the FTIR analysis. For the citric acid-treated KBC, the increase in the O/C was intermediate between those observed for KBC-H_2_O_2_ and KBC-NaClO, showing a slight cleansing effect on KBC. In this case, the increase in the O/C atomic ratio may also be due to the contribution of the COOH groups from citric acid, which could have acted as a crosslinking agent for KBC, by participating in esterification reactions with the OH groups of cellulose [62]. In addition, the theoretical O/C atomic ratio in citric acid is 1.17, higher than that of cellulose.

The high-resolution spectra in Figure 5 may provide further insight into the chemical changes occurring on the surface of KBC following the chemical treatments with different chemical reagents. The C1s region of KBC was fitted with four individual peaks: a first peak (C1) centered at ~285 eV which can be assigned to the C-C, C-H, C=C [63], and C-N bonds [64] from impurities present in KBC including aromatic compounds, peptides, proteins, enzymes, and melanoidins contaminants; a second peak (C2) centered at ~286.7 eV which can be associated with the C-O bonds in the C-OH groups and C-O-C linkages in cellulose [65]; a third peak (C3) at ~288.1 eV which can be attributed to the O-C-O hemiacetal groups and C=O groups in oxidized species [66]; and, finally, a fourth peak at ~289 eV which can be assigned to the O-C=O linkages in the COOH groups [67].

As shown in Table 2, following the NaOH, NaClO, and H_2_O_2_ treatments, the C1 peak contribution decreases significantly, which proves the cleaning action of these agents. Since the decrease in the C1 peak areas occurs concomitantly with an increase in the C2 peak areas, it can be deduced that the higher percentage of C-O groups in the KBC-NaOH, KBC-NaClO, and KBC-H_2_O_2_ samples comes from the cellulose in these samples becoming more exposed due to the removal of non-cellulosic impurities from its surface [68].

For the H_2_SO_4_ treatment, the decrease in the C1 peak contribution was smaller, showing the inferior cleaning capacity of H_2_SO_4_ as compared to NaOH, NaClO, and H_2_O_2_, which is in line with the FTIR results. Treatment with citric acid caused an even smaller decrease in the contribution of the C1 peak specific to impurities, which hints at a reduced cleaning ability of citric acid, in agreement with the FTIR data. However, in this case, the high percentage of C1 carbon may also come from the contributions of the C-C bonds (C atoms without O neighbors [68]) from the citrate moieties attached to KBC following the esterification with citric acid. Interestingly, regardless of the purification agent, an increase in the relative amount of the C3 carbon atom was observed, especially for the KBC-H_2_O_2_ sample, where the C3 percentage rose from 6% to 14.5%. In this case, the C3/C2 ratio (0.21) becomes close to the theoretical value of 0.2, specific to pure cellulose [28], showing, besides the known oxidizing action of H_2_O_2_ on cellulose, the purification effect of H_2_O_2_ on KBC, which leads to increased exposure of cellulose at the surface of this sample [68].

Finally, the treatments with NaClO and H_2_O_2_ led to an increase in the relative content of C4 (from the O-C=O moieties) in KBC. This could be attributed to the oxidizing effect of H_2_O_2_ and NaClO, which can lead to the partial conversion of the OH groups in cellulose to COOH [52,69]. An increase in the amount of O-C=O groups can also be observed in the case of KBC-citric, probably due to the unreacted COOH groups from the citrate units bound to KBC upon treatment with citric acid. An increase in the area of the C4 XPS peak associated with the O-C=O moieties was also reported by Cui et al. [70] following the esterification of cellulose with citric acid at 130 °C, at a citric acid:cellulose weight ratio of 3:1, and has been attributed both to the free COOH groups from the citrate moieties attached to cellulose, as well as to the new ester bonds formed between citric acid and cellulose.

### 3.3. X-Ray Diffraction

**Figure 6:**
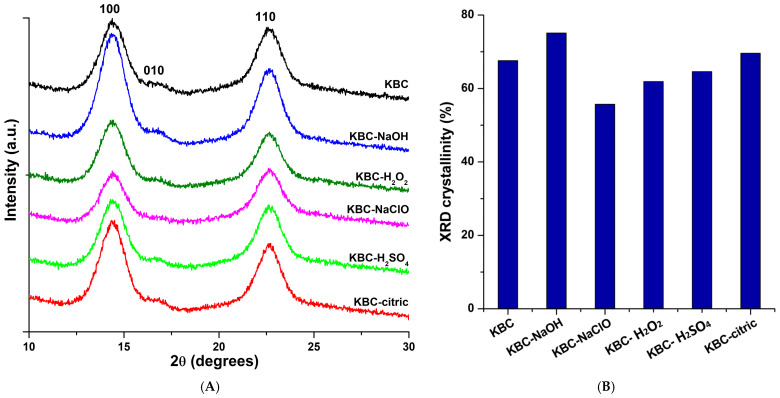
XRD patterns of control KBC and the KBC-NaOH, KBC-NaClO, KBC-H_2_O_2_, KBC-H_2_SO_4_, and KBC-citric samples (**A**); XRD crystallinity of KBC samples (**B**).

Both chemical and mechanical treatments can influence the crystalline structure and the crystallinity of KBC samples. The XRD patterns of KBC samples (Figure 6A) show three peaks at 14.41 ± 0.02°, 16.70 ± 0.05°, and 22.62 ± 0.01° (mean values of all the samples), which correspond to the crystallographic planes of cellulose Iα (100), (010), and (110) [71]. Therefore, the chemical and mechanical treatments did not modify the crystalline structure of KBC, the characteristic peaks showing variations of less than 0.05° between samples. As a type of bacterial cellulose, KBC contains two allomorphs, Iα and Iβ, but the Iα crystal form is in a much higher proportion, of 73–82%, according to some studies [72], or 68–92%, according to others [71]; therefore, only the crystallographic planes of cellulose Iα were noticed in Figure 6.

The interplanar distance changed very little after the treatments, being 6.142 ± 0.006 Å, 5.304 ± 0.018 Å, and 3.926 ± 0.001 Å (mean values of all the samples) for the three crystallographic planes, respectively, while the crystallite size perpendicular to the (110) plan remained around 5.2 nm for all the samples, regardless of the treatment. Similar values have been reported in previous studies for the 2θ corresponding to the main peaks, the interplanar distances, and crystallite size of bacterial cellulose [71,72,73]. However, the literature on the influence of different chemical treatments on the crystalline structure, interplanar distance, and crystal size of bacterial cellulose is rather scarce and sometimes the conclusions are quite different. Previous studies have shown that the crystalline structure of bacterial cellulose was not modified after maleic acid esterification [74] and sulfuric acid hydrolysis [75], but acetylation induced some changes in the crystalline structure of BC, determined mainly by the crystalline disorder generated by the new acetyl groups [76]; alkali treatment modified the crystalline structure of bacterial cellulose when the NaOH concentration increased over 4% [77], while oxidation of urea/NaOH-treated BC with NaClO in the presence of TEMPO and sodium periodate led to a partial destruction of BC’s crystalline structure [78].

The crystallinity (Xc) of KBC was influenced by the applied treatments (Figure 6B). The Xc increased from 67.6% for the control KBC to 75.1% for KBC-NaOH and decreased to 55.7% and 61.9% after treatments with NaClO and H_2_O_2_, probably due to the oxidizing effects of the two, as also indicated by the FTIR and XPS. Both acid treatments, with H_2_SO_4_ and citric acid, led to small changes in the Xc value, which became 64.6% and 69.6%, respectively. Similar behaviors, i.e., higher crystallinity after alkali treatment [77], lower crystallinity after treatment with oxidizing agents [78], and small variation after acid treatment [74,75], were also reported in previous studies. Treatment of KBC with NaClO, H_2_O_2_, and H_2_SO_4_ may cause the disruption of some hydrogen bonding due to oxidation or sulfonation reactions or the formation, under more drastic conditions (strong acid, oxidizing effects), of lower-molecular-weight fractions [75,78], which can lead to a decrease in crystallinity. Although H_2_SO_4_ has a well-known effect of reducing the amorphous content in cellulose, it can also cleave the glycosidic bonds, leading to more water-soluble short-chain products [79]. Thermogravimetric analysis of the KBC samples could yield more information on the effect of H_2_SO_4_ and the other treatments.

### 3.4. Thermogravimetric Analysis

**Figure 7:**
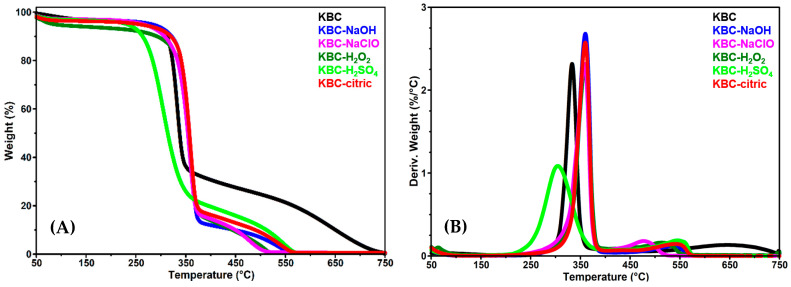
Thermogravimetric (**A**) and derivative thermogravimetric (**B**) curves of control KBC and the KBC-NaOH, KBC-NaClO, KBC-H_2_O_2_, KBC-H_2_SO_4_, and KBC-citric samples.

The thermogravimetric (TG) and derivative thermogravimetric (DTG) curves of control KBC and KBC-NaOH, KBC-NaClO, KBC-H_2_O_2_, KBC-H_2_SO_4_, and KBC-citric samples are shown in Figure 7A,B. The most important thermal parameters, including the onset degradation temperature (T_onset_), determined at the intersection point between the extrapolated baseline and the downward tangent at the steepest point of the weight loss curves, the weight loss at 200 °C (WL_200 °C_), the temperatures at maximum weight loss rates (T_max1_, T_max2_), and residue at 750 °C (R_750 °C_) calculated from the TG and DTG curves are presented in Table 3.

As displayed in Figure 7A,B, control KBC shows three thermal degradation stages: a first step up to 100 °C which can be attributed to the evaporation of water adsorbed or bound in the sample as well as other volatile compounds from KBC [80]; a second step between 220 and 400 °C, where the majority of weight loss takes place due to cellulose degradation through dehydration, depolymerization, and breakdown of the glucose units, resulting in the formation of char residues; and a third, between 400 and 750 °C, which corresponds to the thermal decomposition of the carbonaceous residues formed in the previous step, as well as the breakdown of the degradation compounds resulting from the protein, bacteria, or yeast cell residues embedded in KBC [81,82].

As indicated in Table 3, treatments with NaOH and NaClO led to a rise in the thermal stability of KBC, noticeable by an increase in T_onest_ from 318.0 °C to 339.4 °C and 334.1 °C, respectively, and in the T_max1_ from 333.0 °C to 359.6 °C and 357.6 °C, respectively. This may be attributed to the removal from the KBC membrane of lower-molecular-weight impurities, such as flavonoids, tannins, peptides, and melanoidins, whose thermal degradation starts earlier than that of cellulose. An increase in the T_max_ value from 289 °C to 322 °C and then to 359 °C has been reported before by Gea et al. [83] for a BC that was purified in the first step with a 2.5 wt% NaOH solution and then with a 2.5 wt% NaClO solution.

However, a decrease in the T_max2_ value, associated with the third stage of thermal degradation, from 644.9 °C in KBC to 532.9 °C for KBC-NaOH and 476.9 °C for KBC-NaClO, was observed. This could be attributed to the partial removal from KBC of proteins, lipids, bacteria, and yeast residues, which typically undergo thermal degradation more gradually than cellulose [82]. An almost imperceptible decrease in the WL_200 °C_ of KBC-NaOH and KBC-NaClO as compared to that of KBC can be observed. This could be due to the fact that the thermal degradation of most impurities in the control KBC occurs at temperatures above 200 °C. Treatment with H_2_O_2_ appears to result in a similar improvement in the thermal stability of KBC, noticeable by an increase in the T_onset_ and T_max_ values to 338.3 °C and 359.4 °C, respectively, suggesting that H_2_O_2_ also manifests a purifying action on KBC. Interesting, however, is the higher WL_200 °C_ of KBC-H_2_O_2_ (6.7%) as compared to that of KBC-NaOH (3.4%) and KBC-NaClO (3.2%). As this weight loss occurs mainly below 100 °C, it can be attributed to the evaporation of water from the material. It is possible that the H_2_O_2_ treatment increased the water absorption capacity of KBC [84], due to the newly generated oxidized groups that may have caused the disruption of some of the hydrogen bonding interactions in KBC. Treatment with H_2_SO_4_ led to a significant reduction in the thermal stability of KBC, causing a decrease in the T_onset_ and T_max1_, by 46 °C and 28.7 °C, respectively. This may be due to the acidic hydrolysis of the glycosidic bonds in cellulose, which results in the formation of short chains that are more susceptible to thermal degradation [85] or due to the esterification of some of the OH groups in KBC by H_2_SO_4_, which led to the formation of sulfate half ester (OSO_3_^−^) groups, which are known to catalyze cellulose’s dehydration and accelerate its thermal decomposition [66]. Citric acid treatment also resulted in an increase in the thermal stability of KBC, visible in the significant elevation in the T_onset_ and T_max1_ values by 21.8 °C and 26.4 °C; this is probably due to the known crosslinking effect of citric acid [86]. An increase in the thermal stability (T_onset_ and T_max_) of cellulose nanocrystals (CNCs) after esterification with citric acid was reported by He et al. [87] and was attributed to the substitution of the OH groups in CNCs with the more thermally stable ester groups. All in all, the treatment with different chemical reagents followed by mechanical fibrillation led to cellulosic materials with sufficiently high thermal stability to make them suitable for use in melt-processing operations conducted at relatively elevated temperatures.

### 3.5. Polarized Optical Microscopy

**Figure 8:**
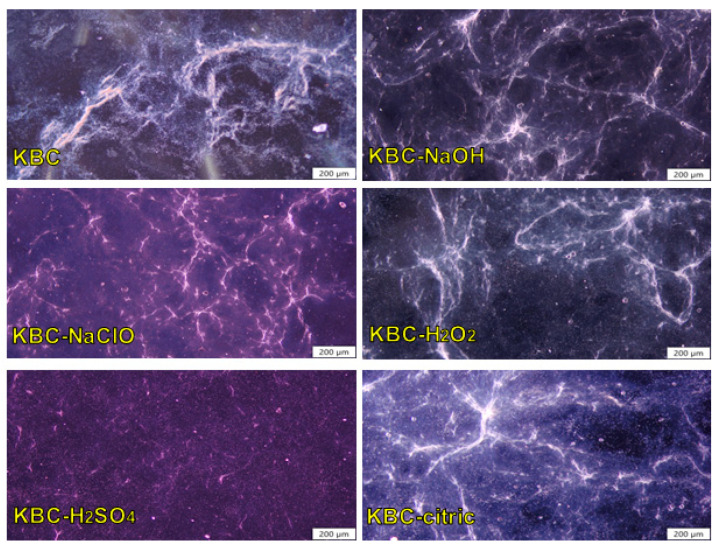
POM images of KBC before and after the chemical treatment with NaOH, NaClO, H_2_O_2_, H_2_SO_4_, and citric acid, respectively, and Ultra-Turrax homogenization.

The optical microscopy images (Figure 8) of the aqueous suspensions of KBC (untreated or previously treated with different chemical reagents) after being subjected to Ultra-Turrax homogenization for 4 min confirmed that this short process resulted in a certain degree of fibrillation of the KBC. Ultra-Turrax homogenization involves the high-speed mixing of a suspension in the narrow gap between a rotor and a stator, followed by its forced expulsion through the stator and circulation back into the mix. The high pressure generated during the process can produce multiple destructive forces (shear, turbulence, cavitation) which can considerably disrupt the hydrogen bonding network in cellulose, resulting in fibrillation and reduction in the fibers’ size [88]. However, in our case, the achieved degree of fibrillation proved to be a result not of the high-speed homogenization alone, but of the combined action of Ultra-Turrax homogenization and the chemical pretreatment of KBC with the aforementioned reagents.

As shown in Figure 8, following high-speed homogenization, the control KBC consists of a 3D network of fibers where clusters and thick bundles of fibers still exist, a sign that advanced fibrillation was not attained in this case. The thick appearance of the fibers could also be caused by the solid impurities from the fermentation medium or bacteria and yeast cell residues that are attached to (or covering) the cellulose fibers [89]. NaOH treatment seems to have facilitated KBC fibrillation and the fiber bundles opening, to a certain extent [90], visible in the detachment of fine fibers from the thick bundles. This may be due to the removal of impurities and the disruption of some of the hydrogen bonding interactions in KBC following the NaOH pretreatment [91], which encouraged the swelling of the cellulose fibers and facilitated their fibrillation during the mechanical treatment. However, thick bundles of fibers can still be noticed in the optical micrographs of KBC-NaOH, suggesting that an advanced degree of fibrillation could not be attained with this treatment alone. Contrastingly, the treatment with NaClO appears to substantially facilitate the fibrillation of KBC, as evidenced by the thinner and more individualized fibers observed in this case. It is important to mention that KBC-NaClO may also contain fractions of finer fibers that could not be visualized by optical microscopy, due to the limitations of the equipment [92].

Ultra-Turrax homogenization of the H_2_O_2_-treated KBC resulted in a moderate level of fibrillation, only slightly superior to that observed in the KBC-NaOH case. Regarding KBC-H_2_O_2_, the achieved degree of fibrillation may be attributed to the partial oxidation of the OH groups in KBC under the action of H_2_O_2_, which led to the disruption of some of the hydrogen bonds in cellulose and the formation of COO^−^ groups that induced electrostatic repulsions between fibers, encouraging their separation during the high-speed homogenization [93]. The most effective route for the fibrillation of KBC appears to be combined treatment with H_2_SO_4_ and homogenization in an Ultra-Turrax, which produced thinner and, possibly, much shorter fibers than in the previous cases, most of them with dimensions below the resolution limit of the optical microscope. This may have been because H_2_SO_4_ attacked the glycosidic bonds in the amorphous regions of cellulose, causing the fragmentation and shortening of the fibers, and also could have led to the conversion of some of the OH groups in cellulose into OSO_3_**^−^** groups [94], which resulted in the disruption of some of the hydrogen bonding interactions in KBC and appearance of electrostatic repulsions between the fibrils, which aided the fibrillation. The Ultra-Turrax homogenization of KBC after chemical treatment with citric acid resulted only in slight fibrillation. This may be due to the limited effectiveness of citric acid in removing the impurities from KBC. These contaminants could have acted as a binder for the fibers, keeping them stuck together, or hindering their swelling and fibrillation [95]. At the same time, due to the multiple COOH groups in its structure, citric acid has the potential to act as a crosslinking agent for cellulose, promoting the linkage of the BC fibrils to each other [86]. These results show the need for an additional defibrillation step, which in this work consisted of microfluidization. The morphological characteristics of the KBC samples after Ultra-Turrax homogenization and microfluidization were evaluated by SEM and AFM.

### 3.6. Scanning Electron Microscopy

**Figure 9:**
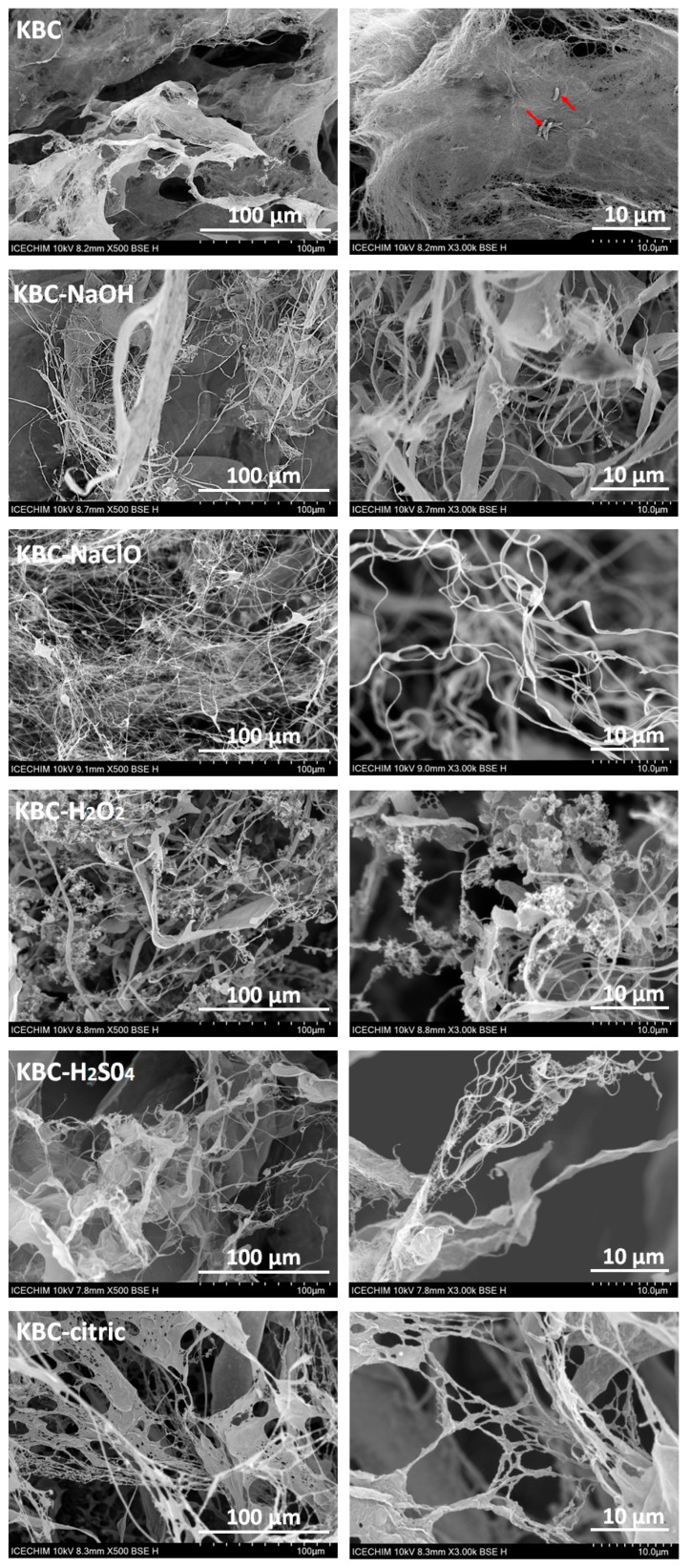
SEM images of KBC after the chemical treatment with NaOH, NaClO, H_2_O_2_, H_2_SO_4_, and citric acid, respectively, Ultra-Turrax homogenization, and microfluidization; bacterial cell debris was marked with arrows in the control KBC image.

The SEM images may provide important information regarding the size and morphology of KBC after the chemical pretreatments and mechanical fibrillation via combined Ultra-Turrax homogenization and microfluidization. As shown in Figure 9, the control KBC sample forms a dense network of interpenetrating fibers, and only slight traces of fibrillation can be observed. Moreover, bacterial cell debris, such as that marked with arrows in Figure 9 (KBC), was frequently observed in the SEM images of unpurified KBC.

In the case of KBC-NaOH, partial fibrillation was observed, evident in the looser structure and the separation of individual nanofibers from the fiber bundles. This demonstrated that the NaOH pretreatment potentiates KBC’s fibrillation through its ability to break hydrogen bonding interactions in KBC [85] and promote swelling of the fibers [86]. For the NaClO treatment, the fibrillation was greatly advanced, giving rise to very thin fibers that assembled into a sparse network. This might be due to the oxidized groups on their surface, as revealed by XPS and FTIR. This probably resulted in the disruption of some of the initial hydrogen bonding interactions in KBC and electrostatic repulsions between the fibers (due to the negatively charged COO^−^ groups), which facilitated KBC’s fibrillation during the mechanical treatments [88]. It should be mentioned that the most advanced fibrillation was observed in this case.

In the case of KBC-H_2_O_2_, fibrillation appears to be similar to or slightly better than that shown by KBC-NaOH, but inferior to that of KBC-NaClO. The pronounced fibrillation observed in the case of KBC-H_2_O_2_ can be attributed to the oxidizing action of H_2_O_2_, which led to the formation of COO^−^ groups in KBC, as indicated by the FTIR and XPS results, thus enhancing fibrillation.

Advanced fibrillation was also obtained for KBC-H_2_SO_4_, where the formation of extremely thin fibers was noticed. This is possibly due to the ability of H_2_SO_4_ to hydrolyze portions in cellulose, weakening KBC’s initial structure, which makes fibrillation easier [89], as well as to the negatively charged OSO_3_^−^ formed on the fibers’ surfaces, which induced electrostatic repulsions between fibers, facilitating the fibrillation [83].

Fibrillation and the formation of a sparse network of nanofibers was also obtained for the KBC-citric sample, although, as shown by the FTIR and XPS results, citric acid did not lead to efficient cleaning of KBC. The high degree of defibrillation may be due to the chemical binding on the cellulose fibers of citrate groups presenting one or two free COO^−^ groups, which led to electrostatic repulsions between the fibers, favoring the fibrillation [90]. However, bundles of fibers and thicker fibers can also be observed in the KBC-citric micrograph, possibly due to the residual impurities binding the fibers together or due to the crosslinking effect of citric acid, which can link the KBC fibrils to each other [76].

### 3.7. Atomic Force Microscopy

**Figure 10:**
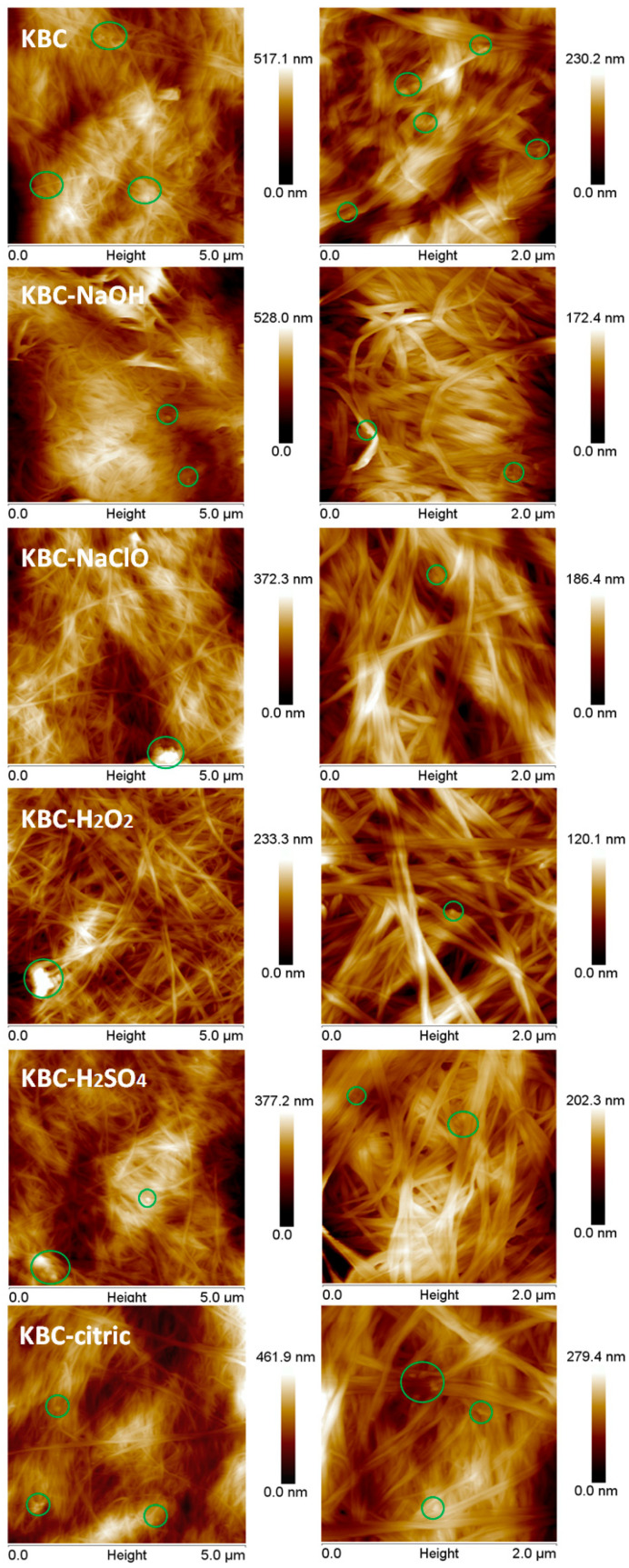
AFM topographic images of KBC before and after the chemical and mechanical treatments; the green circles mark the impurities mostly related to fragmented bacterial cell debris.

The SEM images cannot provide advanced information on the size of the KBC nanofibers formed after the chemical treatments, high-speed homogenization, and microfluidization. AFM investigation of the surface of the KBC film is more suited to emphasizing the changes in the nanofibers’ sizes after the treatments. The width of the ribbons was analyzed using NanoScope 1.20 software (Bruker, Billerica, MA, USA), and the results are shown in Figure 10. As indicated in Figure 9, all the films appear as networks of KBC ribbons, whose width and entanglement differ from sample to sample. Nanoscopic impurities (circled in Figure 10) were also observed in almost all samples, but the most pronounced presence was seen in the case of untreated KBC (control). These impurities may have come from the bacterial cell debris, which was fragmentated into nanometric objects by the mechanical treatments, Ultra-Turrax homogenization, and microfluidization that were applied to the samples.

**Figure 11:**
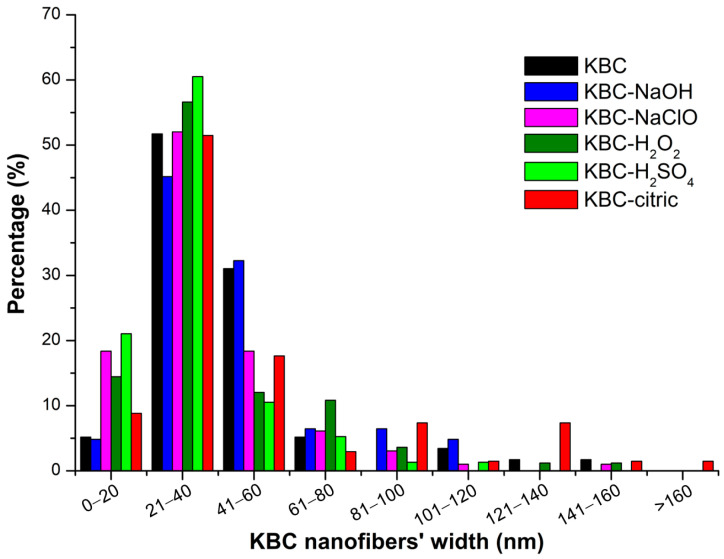
Width distribution of untreated or chemically treated KBC nanofibers after high-speed homogenization and microfluidization, obtained from AFM topographic images.

The nanofiber network of the samples is formed by flat ribbons and individual KBC nanofibers. Individual KBC nanofibers with a width of 10–20 nm appear frequently in the KBC-NaClO, KBC-H_2_O_2_, and KBC-H_2_SO_4_ samples, along with ribbons formed by 2 or 3 nanofibers with a width of 20–40 or 40–60 nm. The proportion of individual nanofibers is high in the case of KBC-H_2_SO_4_ (21.1%), KBC-NaClO (18.4%), and KBC-H_2_O_2_ (14.5%) (Figure 11), showing the great influence of the negatively charged OSO_3_^−^ or COO^−^ groups formed on the fibers’ surfaces by these treatments, which enhance fibrillation by the electrostatic repulsions between fibers. Similar widths were reported for bacterial cellulose nanofibers obtained by the mechanical fibrillation of BC membranes [28]. The largest widths were observed in the case of KBC-citric (Figure 10) due to the influence of the citric acid, which acts as a crosslinking agent, leading to the joining of nanofibers into larger ribbons.

## 4. Conclusions

The Kombucha membrane formed as a by-product during the fermentation of Kombucha tea beverage proved to be an attractive source for the isolation of cellulose nanofibers. In this work, chemical pretreatments with aqueous solutions of NaOH, NaClO, H_2_O_2_, H_2_SO_4_, and citric acid, followed by mechanical fibrillation through Ultra-Turrax homogenization and microfluidization, were applied to isolate cellulose nanofibers from KBC. As shown by the FTIR and XPS analyses, treatments with NaOH, NaClO, and H_2_O_2_ were effective in the purification of KBC, while H_2_SO_4_ and citric acid exhibited a rather weak cleaning action. Moreover, the FTIR and XPS results revealed that the NaClO and H_2_O_2_ treatments resulted in the partial oxidation of KBC, while citric acid participated in esterification and, possibly, crosslinking reactions with KBC.

As a consequence of the advanced removal of impurities from KBC, the treatments with NaOH, NaClO, and H_2_O_2_ also led to an increase in the thermal stability of KBC, reflected by a rise in T_onset_ and T_max_ of more than 16 and 23 °C, respectively. Interestingly, despite the weak purifying effect, citric acid treatment also led to an increase in the T_onset_ and T_max_ of KBC of 21.8 °C and 26.4 °C, probably due to the new ester groups and/or crosslinking bridges formed. At the opposite end, treatment with H_2_SO_4_ led to a severe decrease in the thermal stability of KBC.

Besides their cleaning effect, the applied chemical pretreatments had an important effect on the degree of fibrillation attained. Thus, as indicated by the SEM images, NaOH had a limited effect on promoting KBC fibrillation, while NaClO, H_2_O_2_, H_2_SO_4_, and citric acid strongly encouraged KBC fibrillation, mainly due to the chemical modifications they induced in the KBC’s structure. Altogether, this study represents an important step in obtaining bacterial cellulose nanofibers from an inexpensive and abundant source, commonly regarded as a waste material, with potential applications as reinforcing agents in polymer nanocomposites, adsorbent materials, or scaffolds for tissue regeneration.

## Figures and Tables

**Table 1:** The relative atomic percentages of elements at the surface of the control KBC, KBC-NaOH, KBC-NaClO, KBC-H_2_O_2_, KBC-H_2_SO_4_, and KBC-citric samples.

Sample	Atomic Concentration (%)				O/C
	O 1s	C 1s	Si 2p	N 1s	
KBC	31.9 ± 1.2	66.0 ± 1.1	1.4 ± 0.1	0.7 ± 0.2	0.48 ± 0.03
KBC-NaOH	38.7 ± 1.0	59.9 ± 0.6	0.6 ± 0.3	0.8 ± 0.1	0.65 ± 0.02
KBC-NaClO	36.4 ± 0.6	61.1 ± 0.4	1.0 ± 0.3	1.5 ± 0.3	0.60 ± 0.01
KBC-H_2_O_2_	35.7 ± 2.2	62.2 ± 2.3	0.5 ± 0.4	1.6 ± 0.1	0.58 ± 0.06
KBC-H_2_SO_4_	32.6 ± 1.1	65.2 ± 1.0	1.4 ± 0.2	0.8 ± 0.2	0.50 ± 0.02
KBC-citric	34.2 ± 0.1	62.9 ± 0.4	1.2 ± 0.7	1.7 ± 0.3	0.54 ± 0.00

**Table 2:** The relative atomic concentrations of differently bound carbon atoms at the surface of KBC, KBC-NaOH, KBC-NaClO, KBC-H_2_O_2_, KBC-H_2_SO_4_, and KBC-citric samples.

Sample	Atomic Concentration (%)			
	C1	C2	C3	C4
KBC	34.2	58.6	6.0	1.2
KBC-NaOH	22.3	66.6	9.6	1.5
KBC-NaClO	19.2	67.3	9.6	3.9
KBC-H_2_O_2_	12.0	68.6	14.5	4.9
KBC-H_2_SO_4_	26.0	65.0	7.1	1.9
KBC-citric	31.3	56.7	8.7	3.3

**Table 3:** Parameters determined from the TG and DTG curves for the KBC, KBC-NaOH, KBC-NaClO, KBC-H_2_O_2_, KBC-H_2_SO_4_, and KBC-citric samples.

Sample	T_onset_ (°C)	WL_200 °C_ (%)	T_max1_ (°C)	T_max2_ (°C)	R_750 °C_ (%)
KBC	318.0	3.9	333.0	644.9	0.25
KBC-NaOH	339.4	3.4	359.6	532.9	0.49
KBC-NaClO	334.1	3.2	357.6	476.9	0.66
KBC-H_2_O_2_	338.3	6.7	359.4	513.2	0.00
KBC-H_2_SO_4_	272.0	3.9	304.3	544.8	0.56
KBC-citric	339.8	4.0	359.4	541.4	0.65

## Data Availability

Data are contained within the article.
